# An Unusual Presentation of Cardiofaciocutaneous Syndrome Diagnosed Through Whole Genome Sequencing: A Case Report

**DOI:** 10.7759/cureus.35021

**Published:** 2023-02-15

**Authors:** Rouzy Alhalak, Mohammed H Al-Haideri, Arif Khan

**Affiliations:** 1 Medicine, Mohammed Bin Rashid University of Medicine and Health Sciences, Dubai, ARE; 2 Pediatric Neurology, Neuropedia Hospital, Dubai, ARE; 3 Pediatric Neurology, Mohammed Bin Rashid University of Medicine and Health Sciences, Dubai, ARE

**Keywords:** developmental delay, rasopathies, costello syndrome, noonan's syndrome, congenital heart disease, cardiofaciocutaneous syndrome, genetic testing

## Abstract

Cardiofaciocutaneous syndrome is a rare, sporadic disease caused by germline mutations in the Ras/MAPK (mitogen-activated protein kinase) pathway. Patients usually present with craniofacial anomalies, cardiac defects, and neurocutaneous abnormalities. The features of cardiofaciocutaneous syndrome overlap with two other syndromes known as Noonan’s syndrome and Costello’s syndrome. Similarly, those two syndromes are caused by mutations in the Ras/MAPK pathway. The diagnosis of cardiofaciocutaneous syndrome is suspected based on the clinical presentation and confirmed by genetic analysis. We report a case of a seven-month-old boy who presented with complaints of developmental delay, poor weight gain, and seizures. Physical examination revealed several dysmorphic features, including coarse facies, long philtrum, thin upper lip, a broad forehead, and long toes. Neurological examination showed hypotonia in all four limbs, with normal power and reflexes. However, the infant did not have any remarkable cutaneous abnormalities. Whole-exome sequencing picked up a *BRAF* gene mutation, and the patient was diagnosed with cardiofaciocutaneous syndrome. On follow-up, the patient developed findings suggestive of autoimmune hepatitis. Cardiofaciocutaneous syndrome remains a challenging diagnosis that requires a detailed assessment of the patient, as well as qualified centers with genetic analysis for diagnosis confirmation. Management of cardiofaciocutaneous patients requires a multidisciplinary team approach in order to improve the outcomes. Further exploration is required into atypical presentations of the disease as well as autoimmune disease associated with RASopathies.

## Introduction

Cardiofaciocutaneous (CFC) syndrome is a sporadic disorder that presents with developmental delay along with other characteristic features, including craniofacial anomalies, cardiac defects, and neurocutaneous abnormalities [1]. It is one of the RASopathies, a group of syndromes that result from germline mutations involving genes in the Ras/mitogen-activated protein kinase (MAPK) pathway [2]. The majority of cases are caused by a gain of function mutation of the *BRAF* gene, which is part of the Ras/MAPK pathway [3]. Other genes that have been reported to cause CFC syndrome include KRAS, mitogen-activated protein kinase MEK 1, and MEK 2 [4]. The syndrome was first described in 1986 by Reynolds et al. based on the observation of eight children with developmental anomalies [5]. Since then, around 400 cases have been reported. CFC syndrome overlaps clinically with two other RASopathies known as Noonan’s syndrome and Costello’s syndrome, both caused by mutations involving the Ras/ERK pathway [6]. The prevalence of the syndrome worldwide remains unknown; a study conducted in Japan estimated the prevalence of CFC syndrome to be 1/810,000 [7]. However, the prevalence is believed to be higher. Recognition of this syndrome and its presentation is essential in guiding future genetic counseling and directing patient management. In this article, we present a case of CFC syndrome diagnosed by whole genome sequencing. The patient in our study had an unusual absence of dermatological features that are commonly seen in patients with CFC syndrome. Furthermore, this is the first reported case of autoimmune hepatitis in CFC syndrome.

## Case presentation

A seven-month-old boy born of second-degree consanguinity was brought to our clinic with complaints of developmental delay. According to the parents, the child was not smiling or expressing any reactions in response to stimulus. He was not able to eat solid food by six months. He achieved head control at four months. The parents also reported that their child was not gaining weight and that he experienced neonatal seizures.

**Figure 1:**
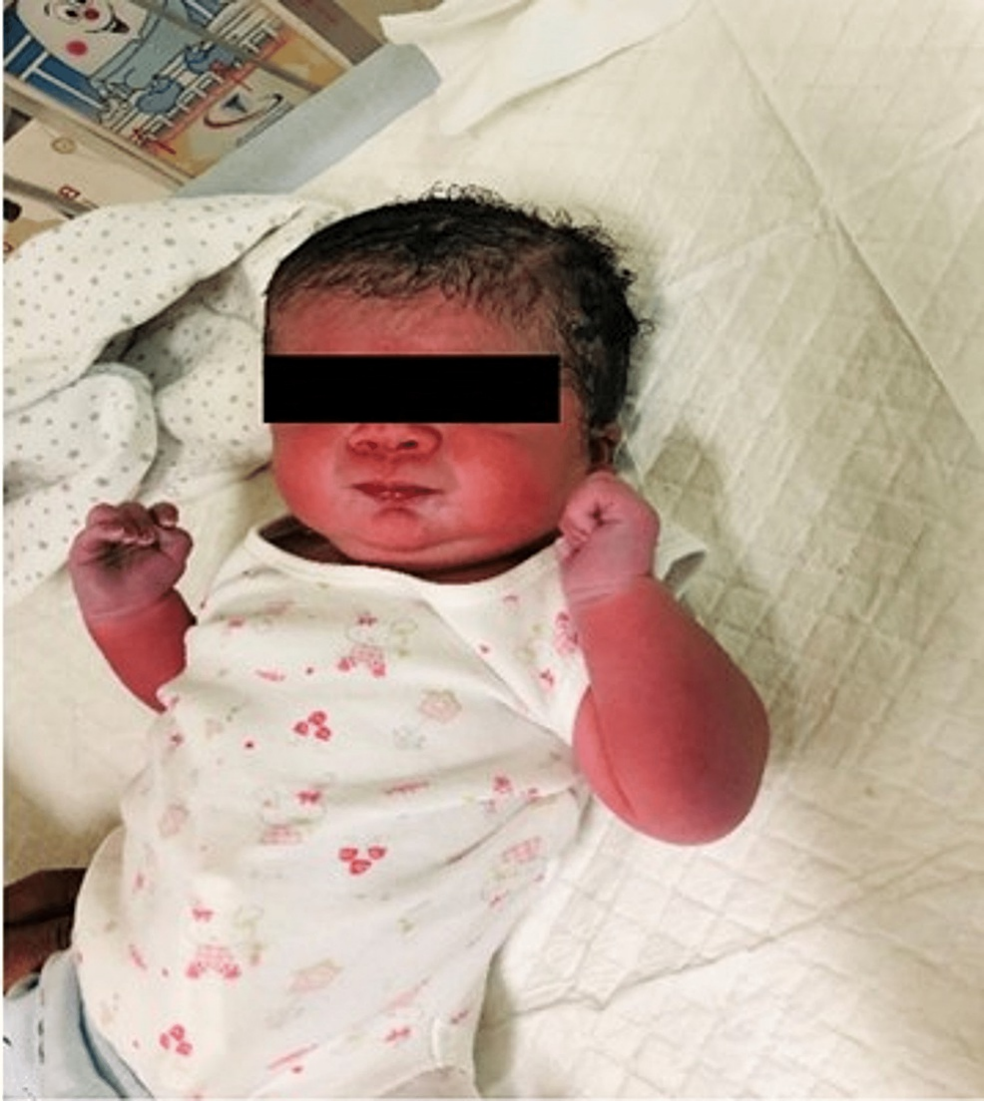
Patient after birth. No cutaneous or dysmorphic features were noted.

Antenatal history was significant for gestational diabetes managed with insulin. The mother had an obstetric history of two previous miscarriages during the early weeks of pregnancy. The child was a late pre-term, he had an uneventful cesarean delivery, and his birth weight was 3.9 kg (Figure 1). Following delivery, the baby developed respiratory distress, hypocalcemia, and hypoglycemia, which mandated neonatal intensive care unit (NICU) admission for five days. Echocardiography performed in the NICU showed small patent ductus arteriosus with patent foramen ovale and diabetic hypertrophic cardiomyopathy.

The patient is the youngest of three children, born to a 40-year-old mother and a 42-year-old father. Both older siblings are healthy and have no developmental concerns. There is no reported family history of mental, developmental, or neurocutaneous diseases.

Detailed head-to-toe examination of the patient revealed several dysmorphic features including coarse facies, long philtrum, thin upper lip, a broad forehead, and long toes. Neurological examination showed hypotonia in all four limbs, with normal power and reflexes. On general inspection of the skin, there were no remarkable cutaneous abnormalities; the patient had normal male genitalia and no organomegaly.

Routine blood investigations including full blood count, liver function test, thyroid function test, 17-hydroxyprogesterone, and urinalysis were performed, which were all within normal limits. Karyotyping showed a genetically male child (46XY). The patient was diagnosed with CFC syndrome by whole exome sequencing which picked up a *BRAF* gene mutation. The *BRAF* variant c1495A>G p.(Lys499Glu) causes an amino acid change from Lys to Glu at position 499; this variant has previously been described as disease-causing in CFC syndrome (Table 1).

**Table 1:** The BRAF variant c1495A>G p.(Lys499Glu) causes an amino acid change from Lys to Glu at position 499; this variant has previously been described as disease-causing in cardiofaciocutaneous syndrome.

Gene	Variant coordinates	Zygosity	In silico parameters	Allele frequencies	Type and classification
*BRAF*	Chr7(GRCh37):g.140477813T>C NM_004333.4:c. 1495A>G p.(Lys499Glu) Exon 12	Het	PolyPhen: benign Align-GVGD: C55 SIFT: deleterious Mutation taster: disease causing Conservation: nt high/aa high	gnomA D: - ESP: - 1000 G: - CentoMD: 0.000022	Missense pathogenic (class 1)

The patient was discharged with the appropriate counseling regarding the need for physiotherapy sessions to improve his strength and posture, a dietician to provide him with adequate nutritional help, and a cardiologist to guide him through the appropriate follow-up plan regarding the echocardiography findings.

At one year of age, the patient started developing weight loss, jaundice, and scleral icterus. Work-up revealed elevated liver enzymes (ALT of 1,728 U/L, AST of 2,931.2 U/L, direct bilirubin of 8.9 µmol/L, and ALP of 314 U/L), as well as positive anti-LKM-1 (liver- kidney-microsomal antibodies-1). This was suggestive of autoimmune hepatitis type II. A biopsy of the right lobe of the liver showed acute cholestatic hepatitis, mild ductular proliferation, hepatocellular glycogenation, and focal syncytial giant cell pattern. The patient was then started on azathioprine, which led to clinical improvement as well as decrease in disease activity index on laboratory studies.

The patient was last seen at 2 years and 7 months of age. During the consultation, the patient demonstrated signs of severe developmental delay. These signs include the inability to speak, walk, follow simple instructions, display gestures, or understand emotions. Furthermore, the patient developed nystagmus (exotropia), esophageal reflux, and poor oromotor functions. The patient is currently receiving care under a specialized multidisciplinary team in a tertiary hospital.

## Discussion

CFC syndrome remains a rare entity that is not commonly encountered in clinical practice. Hence, symptoms of each reported case are broken down individually. A few case reports from existing literature revealed similar findings to this case, which include dysmorphic features of the face and hypotonia in all four extremities. These symptoms were commonly accompanied by moderate-to-severe developmental delay in all four domains. Most of the reported cases presented with unmet motor milestones; the children were not able to sit, stand, or walk without support at the ages they were expected to [8]. Poor height and weight gain was also noted in a few reported cases [9]. Furthermore, cardiac manifestations such as atrial septal defects, ventricular septal defects, pulmonary valve stenosis, and hypertrophic cardiomyopathy have all been reported and are supplemental to the diagnosis of CFC syndrome [4]. This case, however, has a remarkable absence of any dermatological abnormalities, which are one of the hallmarks of CFC syndrome, hence the name. Keratosis pilaris and ichthyosis were the most commonly encountered skin abnormalities. Sparse hair on the head and eyebrows, nail dystrophy, and non-seborrheic eczema were also common findings in previously reported cases of CFC syndrome [4]. Moreover, xerosis, hyperkeratosis, ulerythema ophryogenes, pigmented moles, and hemangiomas were all described in the literature [10]. In addition, consanguineous marriage of the parents was an evident pattern seen in many of these cases including this case. Birth and perinatal complications such as respiratory distress and electrolytes abnormalities were also present in many diagnosed cases of CFC syndrome. On the contrary, seizures were only present in one other reported case in the literature at the time of writing this paper [11].

CFC syndrome is part of a family of genetic disorders that involve the Ras/RAF/MEK/ERK signaling pathways [12]. Other syndromes that belong to this group include Noonan’s syndrome, which is usually challenging to distinguish from CFC syndrome. In addition to Costello’s syndrome, which has multiple features in common with CFC syndrome and Noonan’s syndrome, such as coarse face, short stature, and cardiac manifestations such as pulmonic stenosis [13]. The differences between these syndromes and their diagnostic criteria have been discussed thoroughly in the literature, numerous case reports and literature reviews were published on the topic, and an index was created in 2002 for the diagnosis of CFC syndrome using a set of phenotypic characteristics and presentations [14]. However, the boundaries between these syndromes remain vague. The diagnosis in this case was made through whole genome sequencing rather than pure clinical assessment.

An association between RASopathies and autoimmune disorders has often been described in the literature. Siano et al. reviewed 69 patients with different RASopathies to investigate the association between autoimmune diseases and RASopathies. They found recurrent upper respiratory tract infections in two patients, alopecia in one patient, and psoriasis in another. Low IgA levels, low CD8 T cells, and anti-tg and tissue-TPO antibodies were also detected in a number of patients. Interestingly, all the tested patients had higher inflammatory molecules compared to controls [15]. Similarly, Quaio et al. analyzed clinical and laboratory features in 42 RASopathy patients; 14% of patients fulfilled the clinical criteria for autoimmune disease [16]. Loddo et al. reported a case of Noonan’s syndrome with autoimmune hepatitis type 1 [17]. In this case, we report the first case of autoimmune hepatitis in CFC syndrome in the literature. Autoimmune liver disease in the context of RASopathies seems to have a pattern of developing in early childhood. Understanding the relationship between the genetic disorder and the autoimmune disease is of paramount importance for recognizing the disease early and intervening appropriately.

## Conclusions

CFC syndrome, among other rare genetic disorders, remains unexplored. This case report highlights the possibility of having the mutated BRAF variant c1495A>G p.(Lys499Glu), without displaying cutaneous abnormalities. The management of such diseases requires a multidisciplinary approach in order to improve quality of life. Keeping an open eye for atypical pathological developments, such as autoimmune disease and seizures, is also important.
